# Predictors of gastrointestinal lesions on endoscopy in iron deficiency anemia without gastrointestinal symptoms

**DOI:** 10.1186/1471-230X-8-52

**Published:** 2008-11-09

**Authors:** Shahid Majid, Mohammad Salih, Rozina Wasaya, Wasim Jafri

**Affiliations:** 1 Section of Gastroenterology, Department of Medicine, Aga Khan University Hospital, Karachi, Pakistan

## Abstract

**Background:**

Iron deficiency anaemia (IDA) due to occult gastrointestinal (GI) blood loss usually remains unnoticed until patient become symptomatic. There is sparse data in IDA patients without gastrointestinal symptoms. This study was designed to find out the frequency and predictors of endoscopic lesions in IDA without gastrointestinal symptoms. Cross-sectional study performed on a convenience sample of consecutive subjects.

**Methods:**

Ninety five consecutive patients with laboratory based diagnosis of IDA having no gastrointestinal symptoms were interviewed and their clinical and biochemical variables were recorded. All the study patients underwent esophago-gastroduodenoscopy (EGD) and colonoscopy. Endoscopic findings were documented as presence/absence of bleeding related lesion and presence/absence of cause of IDA. Multiple logistic regressions were performed to identify variables significantly related to outcome variables.

**Results:**

Possible cause of anaemia was found in 71% and bleeding related lesions were found in 53% of patients. Upper gastrointestinal tract lesions were found in 41% of patients with bleeding related lesions. On multivariable logistic regression; advancing age, low mean corpuscular volume (MCV ≤ 60 fl), and positive fecal occult blood test were predictive factors for bleeding related GI lesions and cause of IDA

**Conclusion:**

Clinical and Biochemical markers can predict gastrointestinal lesions on endoscopy in IDA patients without gastrointestinal symptoms. High proportion of upper gastrointestinal involvement warrants EGD as initial endoscopic procedure however, this needs validation by further studies.

## Introduction

Anaemia is common among general population in developing Asian countries. Iron deficiency is a common cause of anaemia either due to poor intake or chronic blood loss. Iron deficiency anaemia is usually due to chronic gastrointestinal (GI) blood loss when there is no obvious source of bleeding. The standard of care for these patients with IDA includes evaluation of the Gastrointestinal (GI) tract for bleeding lesions [1].

Iron deficiency anemia is considered as an alarm sign for the presence of possible GI malignancies, and inadequate evaluation of patients with IDA may delay the diagnosis of GI tumors especially colorectal cancer [2]. In 20% of patients with IDA a routine upper and lower GI endoscopy may not ascertain GI cause during hospital admission [3].

The available literature, in heterogeneous groups including old age patients and postmenopausal women with IDA, has shown GI lesions in 40 – 70% [4-6]. Studies have shown that increasing age, male gender, ferritin level, prior NSAIDs use, positive fecal occult blood test were factors predictors of endoscopic lesions in patients with IDA with and without GI symptoms [7-11]. Studies have concluded that prevalence of endoscopic lesions in patients with IDA without GI symptoms is between 48 – 71%, [9-12] however there is a sparse data related to factors predicting GI lesions in this group.

Important implications for the recognition of iron deficiency anaemia include diagnosis and correction of underlying causes, most of which are identifiable, by means of conventional upper gastrointestinal endoscopy and colonoscopy [13] however it remains unresolved which procedure should be done first [3].

Many studies have concluded that on evaluation of Gastrointestinal Tract for IDA; most of the lesions were in lower GI Tract and have recommended that evaluation for IDA should be started with lower GI examination [14-16].

There is scanty data to predict the nature and site of GI lesions in IDA patients without gastrointestinal symptoms. Therefore there is a need for studies especially from developing asian countries, which may establish endoscopic findings and their predictors in this group.

Primary aim of the study was to identify the predictors of gastrointestinal lesions diagnosed endoscopically in patients with iron deficiency anemia without gastrointestinal symptoms.

## Methods

This Cross-sectional study performed on a convenience sample of consecutive subjects, conducted from May 2006 to August 2007 at Aga Khan University Hospital.

### Inclusion criteria [17]

Iron deficiency anemia was defined as hemoglobin concentration ≤ 12.5 g/dl for men (normal range, 13.5 to 17.5) and ≤ 10.6 g/dl for women (normal range, 11.6 to 15.8) with at least one of the following laboratory values consistent with iron deficiency: a serum iron concentration ≤ 45 μg/dl (normal range 50 to 150) with a transferrin saturation ≤ 10 percent (normal range 16 to 60 percent), serum total iron binding capacity (TIBC) of ≥ 400 μg/dl (Normal range 250 – 400) a serum ferritin concentration ≤ 20 ng/ml for men (normal range 20 to 450) and ≤ 10 ng/ml for women (normal range, 10 to 250).

### Exclusion Criteria

were: i) active bleeding (active GI loss, epistaxis, menorrhagia, heavy menstrual blood loss ii) History of pica iii) Not willing to consent for EGD and Colonoscopy iv) Coagulation disorder v) Thalasemia minor v) Patients with GI symptoms having IDA which included dysphagia, odynophagia, heartburn, vomiting, anorexia, hematochezia, change in bowel habits, unexplained diarrhea, new onset constipation (within last 6 months), and localized abdominal pain.

Eligible subjects, already enlisted for EGD and colonoscopy for Iron deficiency anemia without gastrointestinal symptoms, referred from out patient clinic, admitted in ward or seen as a consult by gastroenterology service in Aga Khan University Hospital, Karachi were enrolled after informed consent.

Detailed history including leading questions for GI symptoms and physical examination were carried out. Baseline investigations including hemoglobin, total leucocyte count, platelets with peripheral blood film, iron studies including: serum iron, total iron binding capacity (TIBC), transferrin saturation and ferritin were done in all patients. All patients were subjected to fecal occult blood testing before endoscopy by using Hemoccult II slides (SmithKline, Sunnyvale, California.).

Endoscopic procedures were performed in left lateral position to prevent chances of aspiration and blood pressure, heart rate along with oxygen saturation were monitored every 5 minutes using cardiac monitors. All procedures were done under conscious sedation with intravenous midazolam and intravenous fentanyl.

All patients underwent EGD and colonoscopy with cecal intubation. Bleeding related endoscopic lesions were biopsied and duodenal and gastric biopsies were taken if no lesions were found on endoscopy. All upper GI endocopies were performed with Olympus GIF-Q 160 video scope and colonoscopies were performed with Pentax 3500 EC-3840LK video scope.

Following lesions were considered as source of IDA on UGI endoscopy: Esophagitis with erosions involving at least 5 mm of the mucosal surface of the esophagus, gastric and duodenal ulcers (>0.5 cm in diameter), carcinoma, adenomatous polyps (>0.5 cm in diameter), 5 or more vascular ectasias [18], erosive gastritis or duodenitis (defined as multiple mucosal defects encircled by erythema), and hiatal hernia with Cameron's erosions, portal hypertensive gastropathy and esophago-gastric varices. Non-bleeding causes of Iron deficiency anemia included the following: histopathologically proven celiac disease [18]. Helicobacter pylori associated chronic gastritis and atrophic gastritis were considered as a possible cause of IDA only when all other causes were excluded [8,11,18].

**Bleeding related lesions **were defined as obvious lesions on endoscopy which can cause anaemia. **Cause of anaemia **defined as patients with bleeding related lesions and patients with no obvious bleeding related lesions on endoscopy but histopathology consistent with possible cause for anaemia which included helicobacter pylori gastritis, atrophic gastritis and celiac disease.

Following lesions were considered as source of IDA on colonoscopy: colonic mass, one or more polyps (> 0.5 cm in diameter), 5 or more vascular ectasias, a vascular ectasia greater than 0.7 cm in size, colonic ulcer(s), and histopathologically proven inflammatory bowel disease [18]. Diffuse diverticular disease was considered as a possible cause of IDA only in those patients with no other causes of blood loss on upper and lower GI endoscopies [11].

### Dependent and Independent variables

Endoscopic lesions including bleeding and non bleeding causes for IDA were classified according to following outcome variables i) Absence or presence of bleeding related lesion ii) Absence/presence of cause of IDA (including bleeding and non bleeding causes).

Following **independent variables **were investigated for each outcome variable age (years), gender (male/female), hemoglobin level (gm/dl), MCV (fl), serum iron, feacal occult blood (positive/negative), family history of cancer (yes/no), weight loss (yes/no), prior therapy for IDA (yes/no), drug history (NSAIDs [yes/no].

### Data Analysis Procedure

Data entered and then analyzed on software Statistical Packages for Social Sciences (SPSS, Chicago, IL) version 15.

Mean ± standard deviation were computed for continuous variables of age, hemoglobin, white blood cells, platelets, MCV, serum ferritin level, serum iron, serum TIBC, transferrin saturation. Frequency and percentages were calculated for categorical data like male and female ratio, drug history, weight loss, family history of GI cancers, prior therapy for IDA, fecal occult blood, gastrointestinal lesions.

Univariate analysis was performed by using simple logistic regression for association between independent and outcome variables. A p-value ≤ 0.05 was considered statistically significant. All the variables with p-value ≤ 0.25 were included in multiple logistic regression analysis. Multivariable logistic regression was used to predict gastrointestinal lesions with clinical and biochemical variables.

## Results

Two hundred fifty patients underwent endoscopy for iron deficiency anaemia with and without gastrointestinal symptoms during the study period. Ninety five patients met the inclusion criteria. Table 1 shows demographic and biochemical characteristics of patients

**Table 1:** Demographic characteristics and biochemical variables of patients with asymptomatic iron deficiency anaemia (n = 95)

*Gender Male/Female*	48/47
*Age (yrs)*	52.1(16.8)
*NSAID users*	22 (23.2%)
*Hx of Weight loss*	29 (30.5%)
*Hx of Fatigue/Lethargy*	18 (18.9%)
*Family Hx of GI cancers*	02 (2.1%)
*Prior Hx of IDA therapy*	09 (9.5%)
*Hemoglobin (Hb) (gm/dl)*	7.1(2.0)
*Mean corpuscular volume (MCV) (fl)*	61.2(5.6)
*Serum Iron (μg/dl)*	11.5(6.1)
*TIBC (μg/dl)*	388(84)
*Transferrin saturation (%)*	4.3(2.5)
*Ferritin (ng/ml)*	6.1(3.8)
*Feacal Occult Blood (FOB) Positive*	30 (31.6%)

**Figure 1:**
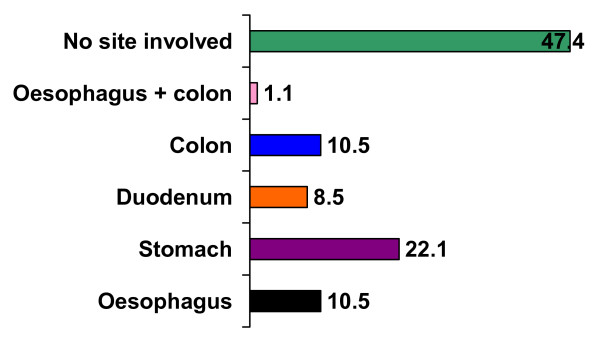
Site of Involvement of gastrointestinal tract (Percentage).

Out of ninety five patients endoscopic lesions were found in 50 (52.6%) of patients. Endoscopic lesions were predominantly in upper gastrointestinal tract. Figure 1 shows site of endoscopic lesions in gastrointestinal tract.

Seventeen patients had no obvious endoscopic lesion but on histopathological examination had either helicobacter associated gastritis or celiac disease, the later was confirmed on serologic testing. Table 2 shows etiology for the cause of iron deficiency anaemia in patients without gastrointestinal symptoms.

**Table 2:** Etiology for iron deficiency anaemia without gastrointestinal symptoms

**Bleeding Lesions**	**No (%)**
***Upper gastrointestinal Lesions***	
Hiatal Hernia with Cameron Lesion	2 (2.1)
Erosive oesophagitis	6 (6.3)
¶Oesophageal Mass	01(1.1)
Erosive Gastritis	08(8.4)
Gastric Ulcer	05(5.3)
‡Gastric Mass	03(3.2)
†Gastric polyp	04(4.2)
Hypertensive Portal Gastropathy	02(2.1)
Erosive Duodenitis	03(3.2)
Duodenal ulcer	05(5.3)
	
***Lower Gastrointestinal Lesion***	
Colonic Ulcer	04(4.3)
††Colonic Mass	02(2.1)
*Colonic Polyp	02(2.1)
Vascular Ectasia in Colon	01(1.1)
Diffuse Diverticular Disease	01(1.1)
	
***Both Upper and Lower gastrointestinal lesion***	
Erosive Oesophagitis + Colonic ulceration	01(1.1)
***Non Bleeding Lesions***	
Helicobacter related Gastritis	14(14.7)
Celiac Disease	03(3.2)
***No Endoscopic or Histopathologic lesion***	27 (28.4)

¶Adenocarcinoma, ‡2 were adenocarcinoma, one lymphoma †one GIST, 3 hyperplastic polp, †† adenocarcinoma, * hyperplastic polyp.

In upper gastrointestinal tract five patients had malignancy out of which one had oesophageal adenocarcinoma, two had gastric adenocarcinoma, one had gastric lymphoma and one had gastrointestinal stromal tumor (GIST). In lower gastrointestinal tract three patients had ulcerative colitis, two had colonic carcinoma and two had colonic tuberculosis with involvement of cecum and terminal ileum.

On univariate analysis age (p < 0.001), hemoglobin (p = 0.04), MCV ≤ 60 fl (p = 0.003), hemoglobin ≤ 9 gm/dl (p = 0.004), FOB (p < 0.001) and NSAID use (p = 0.04) were significantly related to bleeding related endoscopic lesions (Table 3, 4). On multivariable logistic regression analysis MCV ≤ 60 fl (OR 3.5, 95%CI 1.2 – 10.0), FOB (OR 16.4, 95% CI 3.9 – 67.3) and age (OR 1.05, 95% CI 1.02 – 1.07) were found independent predictors for bleeding related endoscopic lesions in patients with IDA without gastrointestinal symptoms.

**Table 3:** Univariate analysis: Clinical and biochemical factors related to bleeding lesions on endoscopy

	**Endoscopic lesions Identified (n = 51)**	**No endoscopic lesions Identified (n = 44)**	**Odds ratio**	**95%CI**	**P value**
***Clinical variables***					
Age (yrs) Mean (SD)	57.3(14.5)	49.3(17.4)	1.04	1.01 – 1.07	<0.001
Age ≥ 55 yrs †	30(58.8%)	10 (22.7%)	4.8	1.9 – 11.9	<0.001
NSAID users ††	16(31.4%)	06(13.6%)	2.8	1.0 – 8.2	0.04
Hx of Weight loss †††	16(31.4%)	13(29.5%)	1.0	0.4 – 2.6	0.84
Hx of Fatigue/Lethargy ¶	13(25.5%)	05(11.4%)	2.6	0.8 – 8.2	0.11
Prior Hx of IDA therapy ¶¶	07(13.7%)	02(4.5%)	3.3	0.6 – 0.9	0.14
					
***Biochemical Variables***					
Hb≤ (gm/dl) Mean (SD)	6.6(1.9)	7.5(2.1)	0.8	0.6 – 0.9	0.04
Hb ≤ 9 gm/dl ‡	46(90.2%)	29(65.9%)	4.7	1.5 – 14.4	0.004
MCV (fl) Mean (SD)	60.3(5.6)	62.2(5.6)	0.9	0.8 – 1.0	0.1
MCV ≤ 60 fl ‡‡	32(62.7%)	14(31.8%)	3.6	1.5 – 8.4	0.003
Serum Iron (μg/dl) Mean(SD)	10.5(6.8)	12.5(5.1)	0.9	0.8 – 1.0 0.13	
Serum Iron ≤ 10 (μg/dl) *	31(60%)	19(43.0%)	2.0	0.8 – 4.6 0.08	
TIBC (μg/dl) Mean (SD)	396(88)	379(79)	1.0	0.9 – 1.0 0.33	
Transferrin saturation (%) Mean (SD)	4.4(4.4)	4.1(4.1)	1.0	0.9 – 1.2 0.47	
Ferritin (ng/ml) Mean (SD)	6.3(4.2)	5.8(3.3)	1.03	0.9 – 1.1 0.54	
Ferritin ≤ 5 (ng/ml) **	28(54.9%)	18(40.9%)	1.75	0.7 – 3.9 0.17	
FOB (Positive) ***	27(52.9%)	03(6.8%)	15.3	4.2 – 56.1	<0.001

Reference groups: †Age <55 yrs; ††No NSAIDS use; ††† No Hx of weight loss; ¶No Hx of Fatigue/Lethargy; ¶¶ No Prior Hx of IDA therapy; **‡ **Hb > 9 gm/dl; **‡‡ **MCV>60 fl; * Serum Iron > 10 (μg/dl); ** Ferritin> 5 (ng/ml); *** FOB (Negative)

**Table 4:** Multivariable Logistic regression analysis; independent predicators of bleeding related endoscopic lesion

**Variable**	**P value**	**Regression coefficients (SE)**	**Adjusted odds ratio**	**95% Confidence Interval**
Age (Years)	0.002	0.05 (0.01)	1.05	1.02 – 1.07
Fecal Occult Blood Positive	<0.001	2.8 (0.72)	16.4	3.9 – 67.3
MCV ≤ 60 (fl)	0.01	1.2 (0.53)	3.5	1.2 – 10.0

For cause of iron deficiency anemia which included bleeding and non bleeding related etiologies; age (p = 0.004), MCV ≤ 60 fl (p < 0.001), hemoglobin ≤ 9 gm/dl (p = 0.06), TIBC (μg/dl) (p = 0.04) and positive fecal occult blood (p = 0.001) were significant factors associated with cause of iron deficiency anemia on univariate analysis. On Multivariable logistic regression analysis after adjusting for confounding factors age in years (OR 1.04, 95% CI 1.01 – 1.08), MCV ≤ 60 fl (OR 14.8, 95% CI 3.6 – 60.7), fecal occult blood positive (OR 7.8, 95% CI 1.46 – 41.8) were independent predictors of the cause of iron deficiency anemia endoscopically (Table 5, 6)

**Table 5:** Univariate analysis: Clinical and biochemical factors related to cause of iron deficiency anaemia in patients with out gastrointestinal symptoms

	**Cause of Iron Deficiency Identified (n = 68)**	**Cause of Iron deficiency not identified (n = 27)**	**Odds ratio**	**95% CI**	**P value**
***Clinical variables***					
Age (yrs) Mean (SD)	55(14)	44(20)	1.04	1.01 – 1.07	0.004
Age ≥ 55 yrs †	34(50%)	06(22.2%)	3.5	1.2 – 9.7	0.01
NSAID users ††	18(26.5%)	04(14.8%)	2.0	0.6 – 6.8	0.22
Hx of Weight loss †††	19(27.9%)	10(37.03%)	0.6	0.2 – 1.6	0.38
Hx of Fatigue/Lethargy ¶	16(23.5%)	02(7.4%)	3.8	0.8 – 18	0.08
Prior Hx of IDA therapy ¶¶	08(11.8%)	01(3.7%)	3.4	0.4 – 29.1	0.43
					
***Biochemical Variables***					
Hb (gm/dl) Mean (SD)	6.9(1.9)	7.3(2.2)	0.9	0.7 – 1.1	0.44
Hb ≤ 9 gm/dl ‡	57(83.8%)	18(66.7%)	2.5	0.9 – 7.2	0.06
MCV (fl) Mean (SD)	60.0(5.5)	64.0(4.7)	0.8	0.7 – 0.9	0.002
MCV 60 fl ‡‡	43(63.2%)	03(11.1%)	13.7	3.7 – 50.3	<0.001
Serum Iron (μg/ml) Mean (SD)	11.5(6.5)	11.3(5.1)	1.0	0.9 – 1.0	0.86
Serum Iron ≤ 10 (μg/ml)*	35(51.4%)	15(55.5%)	0.85	0.3 – 2.0	0.71
TIBC (μg/dl) Mean (SD)	399(82)	361(85)	1.0	1.0 – 1.01	0.04
Transferrin saturation (%) Mean (SD)	4.3(2.7)	4.1(2.0)	1.9	0.7 – 4.9	0.67
Ferritin (ng/ml) Mean (SD)	6.0(4.0)	6.1(3.3)	0.99	0.8 – 1.1	0.88
Ferritin ≤ 5 (ng/ml) **	36(52.9%)	10(37.0%)	1.9	0.7 – 4.7	0.17
FOB (Positive) ***	28(41.2%)	02(7.4%)	8.7	1.9 – 39.9	0.001

Reference groups: †Age <55 yrs; ††No NSAIDS use; ††† No Hx of weight loss; ¶No Hx of Fatigue/Lethargy; ¶¶ No Prior Hx of IDA therapy; **‡ **Hb > 9 gm/dl; **‡‡ **MCV>60 fl; * Serum Iron > 10 (μg/dl); ** Ferritin> 5 (ng/ml); *** FOB (Negative)

**Table 6:** Multivariable logistic regression: Predictors for cause of iron deficiency in patients without gastrointestinal symptoms

**Variable**	**P value**	**Regression coefficients (SE)**	**Adjusted odds ratio**	**95% Confidence Interval**
Age (Years)	0.01	0.04 (0.01)	1.04	1.01 – 1.08
Fecal Occult Blood Positive	0.01	2.0 (0.85)	7.8	1.46 – 41.8
MCV ≤ 60(fl)	< 0.001	2.7 (0.71)	14.8	3.65 – 60.7

**Figure 2:**
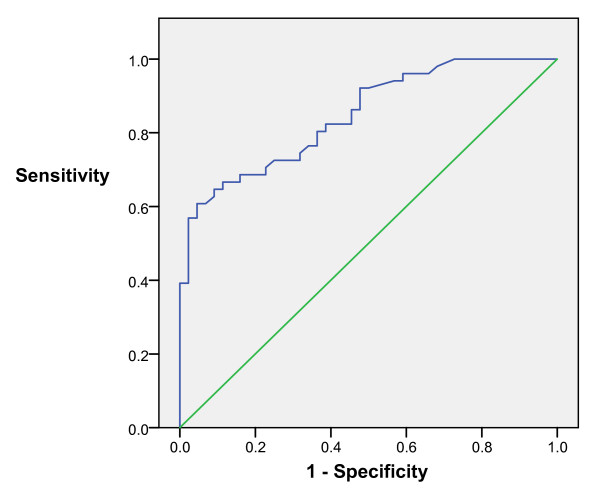
Graph showing ROC curve for bleeding related lesion.

**Figure 3:**
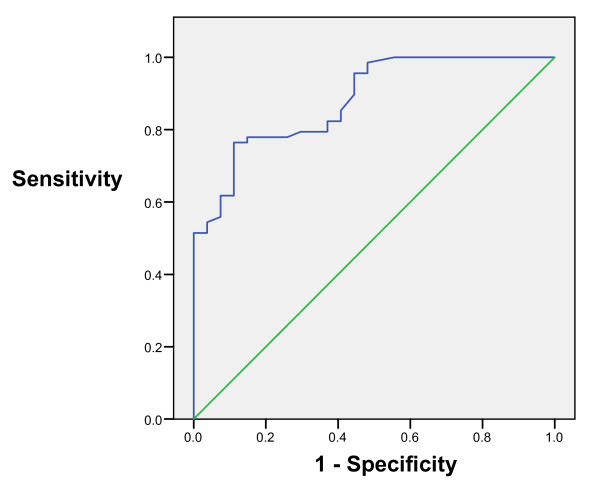
Graph showing ROC curve for cause of iron deficiency anaemia.

Receiver operating characteristics (ROC) analysis for bleeding related lesions showed area under curve 85% with CI 95% between 77 – 92% while ROC analysis for overall cause of anaemia showed area under curve 88% wit 95% CI between 81–95% (Fig 2 and 3).

## Discussion

Iron deficiency anaemia due to chronic blood loss is usually silent and becomes evident when patients become symptomatic [19]. Studies in patients with IDA without gastroenterological symptoms are few and have small number of patients and are in different groups of patients. It is difficult to conclude that what is usual pattern of diseases, factor which can predict the endoscopic out come in IDA patients without gastrointestinal symptoms.

We have scanty data in Asian population regarding endoscopic evaluation of IDA patients especially without gastrointestinal symptoms. Furthermore it is unresolved whether evaluation should be started with upper GI or lower GI endoscopic examination.

Available data in patients with IDA having gastrointestinal symptoms the prevalence of getting endoscopic lesions is up to 70% [4-6] while in asymptomatic IDA patients the cause (bleeding and non-bleeding) was found in 85% [11] and bleeding related lesions were found in 37 – 44% [11,20]. In this study we had 53% of patients with bleeding related cause for IDA and overall cause of IDA (bleeding and non bleeding lesions) was 71% on upper and lower GI endoscopy.

Three patients had celiac disease while14 patients had Helicobacter pylori associated gastritis. It is now recognized that celiac disease can present as IDA without any gastrointestinal symptoms [21,22]. Similarly studies have shown helicobacter pylori infection association with increase in prevalence of IDA [11,18,23].

In this cohort, the frequency of lesions involving lower gastrointestinal tract was low which is striking and contrary to the studies in Caucasian population, in which lower GI lesions were more or equal to upper GI tract [2,9,15]. Another prominent finding in our study was small number of patients had malignancies. Only two patients had colonic malignancy which is again dissimilar to the available data which ranges from 10% to 50% patients [11,15,20,24]. Lower proportion of malignancy in this study may be explained on the basis of lower incidence of gastrointestinal polyps in Asian population [25].

Studies have suggested bidirectional endoscopic evaluation as a workup of IDA and most of them recommended lower gastrointestinal endoscopy first [26,27]. In our study only 10% of patients had colonic involvement while 61% had bleeding and non bleeding causes in upper gastrointestinal tract. Lesions involving both the tracts were present in one patient. Result of this cohort favors that endoscopic evaluation of the upper gastrointestinal tract first.

There is scanty data regarding predictors of endoscopic lesions in IDA patients without GI symptoms, however, hemoglobin, ferritin, female gender and history of NSAIDs have been shown to be associated with endoscopic lesions in patients with IDA having gastrointestinal symptoms [8,10,28]. In the current study there was no correlation with the factors described in patients with GI symptoms. However, our results of advancing age, low mean corpuscular volume and positive fecal occult blood test are in concordance with the available literature of IDA patients.

The main limitation of our study is that we did not investigate patients with negative endoscopies with capsule endoscopy and/or enteroscopy.

In conclusion, patients with iron deficiency anemia without gastrointestinal symptoms obligates comprehensive gastrointestinal tract examination especially with advance age, low mean corpuscular volume and positive fecal occult blood test, as large proportion of patients may have potentially treatable etiology. High proportion of upper gastrointestinal lesions warrants upper gastrointestinal endoscopy as initial endoscopic procedure in patients with iron deficiency anemia with out gastrointestinal symptoms. However these conclusions need validation in further studies.

## Competing interests

The authors declare that they have no competing interests.

## Authors' contributions

SM conceived and designed the study, did literature search, collected and analyzed data. SM drafted the initial manuscript. MS was the coordinator of the study. MS helped in analysis and drafted the final manuscript. RW helped in recruitment of study patients. WJ contributed in designing of the study and supervised the study.

All authors read and approved the final manuscript.

## Pre-publication history

The pre-publication history for this paper can be accessed here:


